# The Efficacy of Remineralizing Materials on Artificial Enamel Lesions: An In Vitro Study

**DOI:** 10.3390/medicina61030462

**Published:** 2025-03-06

**Authors:** Gustė Klimaitė, Arūnas Vasiliauskas, Pranas Grinkevičius, Dominyka Grinkevičienė, Deivydas Šapalas

**Affiliations:** 1 Department of Orthodontics, Faculty of Odontology, Lithuanian University of Health Sciences, Lukšos-Daumanto 6, LT-50161 Kaunas, Lithuania; guste.klimaite@stud.lsmu.lt (G.K.); arunas.vasiliauskas@lsmu.lt (A.V.); 2 Faculty of Odontology, Medical Academy, Lithuanian University of Health Sciences, Eiveniu 2, LT-50161 Kaunas, Lithuania; pranasgrinkevicius@gmail.com; 3 Marine Research Institute, Klaipeda University, Universiteto av. 17, LT-92295 Klaipeda, Lithuania; deivydas.sapalas@ku.lt

**Keywords:** dental caries, dental enamel, tooth remineralization, P11-4 peptide, fluoride varnishes, hydroxyapatites

## Abstract

*Background and Objectives:* Contemporary caries treatment seeks to preserve hard dental tissues as well as to promote lesion remineralization and biological tissue regeneration. While fluoride-based treatments remain the gold standard, their effectiveness has limitations, prompting interest in innovative remineralization technologies. Nano-hydroxyapatite (nano-HA) varnish and self-assembling peptide (SAP) P11-4 are promising biomimetic materials that promote enamel repair, yet long-term data on their efficacy are limited. The objectives of this study were to evaluate the effectiveness of nano-HA varnish and peptide P11-4 in restoring enamel surface hardness after artificial lesions in vitro and to compare them to a control group and fluoride varnish. *Materials and Methods:* Artificial enamel lesions were created on the buccal surfaces of 36 extracted human molars, which were randomly divided into four groups (*n* = 9): control, peptide P11-4, fluoride varnish, and nano-hydroxyapatite varnish. After applying the materials as per manufacturer instructions, specimens were stored in artificial saliva for 14 days. Enamel surface hardness was measured using the Vickers hardness test (HV) at baseline, after demineralization, and after remineralization. Statistical analysis was performed with “IBM SPSS 27.0” using non-parametric Kolmogorov–Smirnov, Kruskal–Wallis, Dunn’s, and Wilcoxon tests. *Results:* The mean baseline enamel hardness value was 323.95 (SD 33.47) HV. After 14 days of demineralization, the mean surface hardness of artificial enamel lesions significantly plummeted to 172.17 (SD 35.96) HV (*p* = 0.000). After 14 days of remineralization, the mean value significantly increased to 213.21 (SD 50.58) HV (*p* = 0.001). The results of the study revealed statistically significant enamel remineralization of the peptide P11-4 group in regard to the demineralized enamel (*p* < 0.05). In contrast, there were no significant results in other treatment groups (*p* > 0.05). Remineralization of enamel was the highest in samples from the P11-4 group (54.1%), followed by the nano-HA group (35.4%), FV group (17.8%), and control group (11.2%). There was a significant difference (*p* < 0.05) in the remineralizing ability between the peptide P11-4 and all other treatment groups. *Conclusions:* Self-assembling peptide P11-4 effectively remineralized artificial enamel lesions and proved to be significantly more effective compared to fluoride varnish and nano-hydroxyapatite varnish, showcasing its superior performance as a remineralizing agent.

## 1. Introduction

Dental caries is a process of tooth hard tissue demineralization caused by acid production from cariogenic bacteria in dental plaque during the metabolism of fermentable carbohydrates [1]. The 2019 Global Burden of Disease Study identified untreated dental caries in permanent teeth as the most common disease globally, affecting approximately two billion people [2]. Caries forms through repeated cycles of demineralization and remineralization [3]. Saliva provides a natural remineralizing effect, but it is often insufficient to protect against caries without additional interventions. Early-stage lesions are reversible, making them ideal for minimally invasive remineralizing treatments, which can halt progression before cavities form.

In recent decades, dental caries treatment has shifted from restorative to non-invasive approaches to delay or avoid restorative procedures. Advances in enamel structure research and biomineralization principles have led to the development of biomimetic treatments that mimic natural mineralization processes [4,5,6]. These treatments aim to stop active caries, shift the balance toward remineralization, and restore the aesthetics, strength, and function of hard tissues [7]. Successful non-invasive treatments reduce reliance on traditional restorations, which often lead to repeated retreatments [8].

Fluoride varnish (FV), a widely used professional remineralizing agent, has long been considered the gold standard in caries prevention [9,10]. However, recent studies suggest its effectiveness has declined [11]. Fluoride primarily integrates into the lesion’s surface zone, with minimal penetration into deeper layers, limiting structural and aesthetic improvements [12]. This limitation has driven the development of new technologies and materials to enhance fluoride’s effects or replace it entirely.

Self-assembling peptide P11-4, also known as oligopeptide 104, is composed of five amino acids: arginine, tryptophan, phenylalanine, glutamine, and glutamic acid [13]. Kind et al. in their study demonstrated that the peptide disperses within an artificially induced enamel lesion, reaching a depth of 70 ± 23 μm, potentially promoting remineralization in deeper subsurface lesions [14]. This claim is further supported by Schmidlin et al., whose experiment with bovine enamel showed recovery of artificial lesions in even deeper layers (200 μm) [15]. Remineralization occurs as calcium and phosphate ions from saliva integrate into the enamel matrix formed by the peptide [16]. The most pronounced effect is observed within the first 30 days after application, resulting in a reduction in lesion size [17]. Performing remineralization procedures at the earliest stage of lesion development may lead to better treatment outcomes [4]. The effect of peptide P11-4 has been studied in various ex vivo and clinical studies [7,16,17,18]. Randomized controlled trials have demonstrated that P11-4 effectively remineralizes early caries lesions on both smooth enamel surfaces [17,19,20,21] and occlusal surfaces [7,22]. The peptide also significantly remineralizes white spot lesions that appear after fixed orthodontic treatment [23,24,25].

Nano-hydroxyapatite (nano-HA) varnish, which can bind to the damaged enamel surface and replace lost hydroxyapatite crystals [26], and peptide P11-4, which can self-assemble into beta-sheets and initiate de novo hydroxyapatite synthesis, are among the latest materials developed for conservative preventive dentistry [18]. Both materials are biologically compatible and safe [7,27], making them widely applicable. However, due to their novelty, there is still a lack of reliable long-term clinical and in vitro studies.

This study aims to evaluate the effects of two innovative remineralizing materials—nano-HA varnish and P11-4 peptide—in vitro, comparing them with a control group and the gold standard, fluoride varnish. The Vickers surface hardness test was used to assess enamel surface hardness changes throughout the study. To our knowledge, this is the first in vitro study to directly compare the efficacy of SAP P11-4 peptide with nano-HA varnish. Our null hypothesis was that there will be no statistically significant differences in the effectiveness of different remineralizing materials in restoring enamel surface hardness.

## 2. Materials and Methods

The research protocol was prepared prior to the study, and ethical approval was obtained from the Bioethics Center of Lithuanian University of Health Sciences (LUHS) on 24 January 2021 (NO. BEC-OF-31).

### 2.1. Sample Size Calculation

An a priori power analysis was conducted using “GIGA calculator” for sample size estimation based on previous research conducted by Savas et al. [28]. With a significance level of α = 0.05 and power = 0.80, the total sample size required was calculated to be 32, with 8 samples in each group. To ensure reliable results, a larger sample size (*n* = 36) was chosen.

### 2.2. Tooth Collection and Enamel Surface Evaluation

For the study, molars extracted at the Face and Jaw Surgery Department of Republic Klaipeda Hospital due to orthodontic, periodontal, or other reasons were collected. Each patient was informed about the study, its procedure, and objectives, and written consent to participate was obtained after completing an anonymous participant information form.

Before the study began, the extracted teeth were stored in a 0.5% chloramine T trihydrate solution (Eurochemicals, Vilnius, Lithuania) at 4 °C. Only intact permanent human molars, unaffected by congenital or acquired diseases, chemicals, or caries (e.g., unfilled, unbleached, and unaltered teeth), were included. Samples with enamel cracks, discoloration, erosion, hypoplasia, or other surface defects were excluded.

The selected teeth (*n* = 36) were transferred to distilled water. Plaque, tartar, and soft tissue residues were mechanically removed and polished using silicon carbide-coated sandpaper (600–2000 grit, Klingspor Schleifsysteme GmbH und Co. KG, Haiger, Germany) and a silicone carbide polishing stone in a straight handpiece (Bien-Air Medical Technologies, Bienne, Switzerland).

Subsequently, using an 18 mm diamond disk with cooling in a straight handpiece, the roots of all extracted teeth were cut 1 mm below the cementoenamel junction. The roots were discarded, and the crowns were used for further analysis. To remove polishing residue, all samples were sprayed with distilled water vapor using the “Hydrovap SG-3.5M” device (Dentalfarm Srl, Turin, Italy) at the Orthodontic Clinic of the Hospital of LUHS.

### 2.3. Sample Preparation and Processing

A specialized tooth holder was designed to immobilize dental crown samples. Using the computer-aided design software “SolidWorks 2021” (Dassault Systèmes, Vélizy-Villacoublay, France), a 3D model of the holder was created. The holder consisted of a cylindrical container with a base, measuring 19.8 mm in diameter and 12 mm in height. The container had a 1 mm thick edge and a depth of 9 mm.

**Figure 1:**
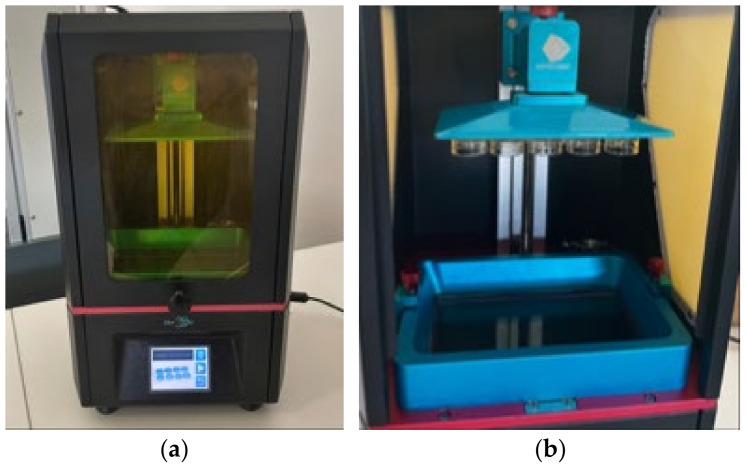
(**a**) “Anycubic Photon” 3D printer; (**b**) forms printed with the printer.

**Figure 2:**
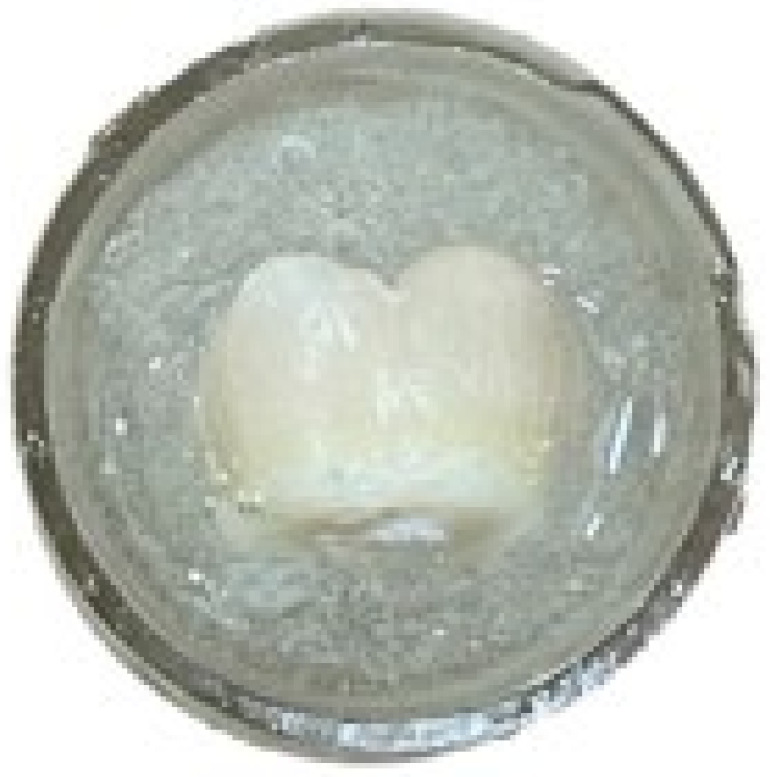
Prepared molar tooth sample.

The holders were fabricated using the “Anycubic Photon” 3D printer (Anycubic, Shenzhen, China) from transparent printing resin, “Anycubic 3D Printing UV Sensitive Resin” (Anycubic, Shenzhen, China), with a UV wavelength of 405 nm and a layer height of 50 μm. The printed molds were cleaned with 95% isopropyl alcohol and cured under UV light to achieve complete polymerization (Figure 1). To secure the sample in the holder, transparent epoxy adhesive (DoneDeal Adhesive Lab. Inc., Hudson, NY, USA) was used. The dental crown was positioned in the center of the holder, aligned with the edge of the mold. Once in place, the sample was heated in a 60 °C chamber to ensure proper fixation (Figure 2).

**Figure 3:**
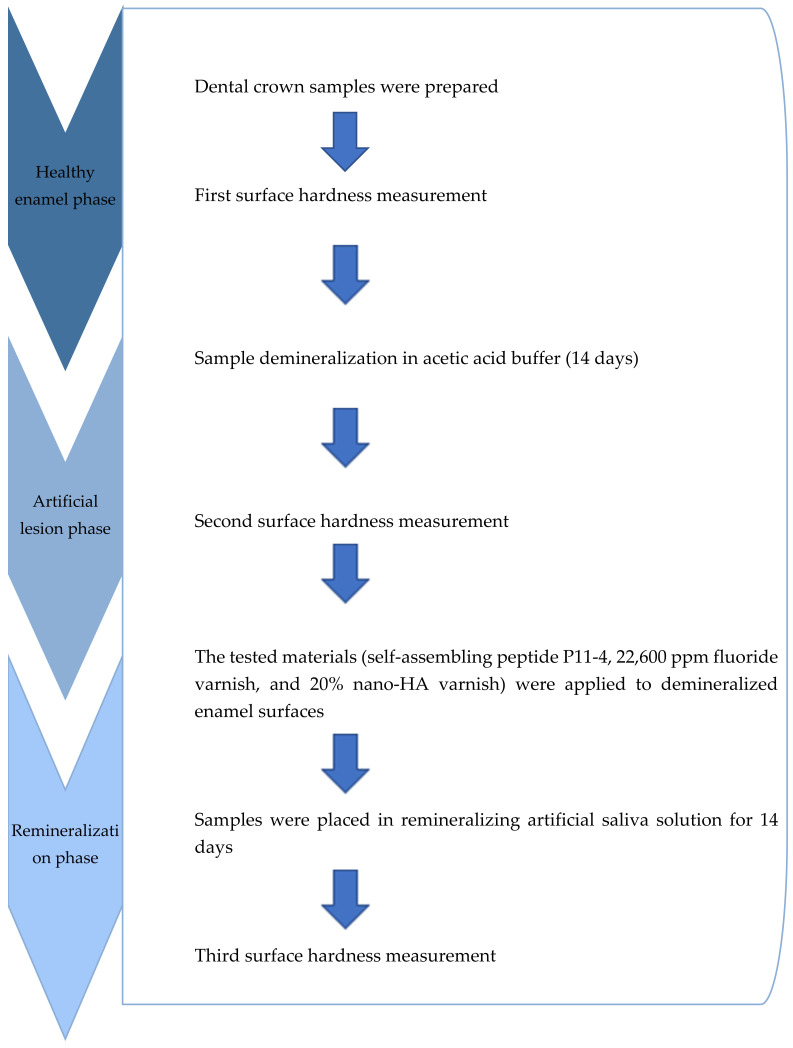
Study methodological diagram.

The study consisted of three main phases: healthy enamel, artificial lesions, and remineralization. The detailed research stages are illustrated in the diagram (Figure 3).

### 2.4. Sample Grouping

A sample (*n* = 36) was randomly divided into four groups (*n* = 9) based on the investigated remineralizing material:

- Group 1: Control group (CG).
- Group 2: Self-assembling peptide P11-4 group (P11-4).
- Group 3: 22,600 ppm fluoride varnish group (FV).
- Group 4: 20% nano-hydroxyapatite varnish group (nano-HA).

The samples were labeled with a permanent marker on the printed container’s base. The first number (I, II, III, IV) indicated the research group, while the second number (1–9) denoted the sample number.

### 2.5. Measurement of Enamel Surface Hardness

**Figure 4:**
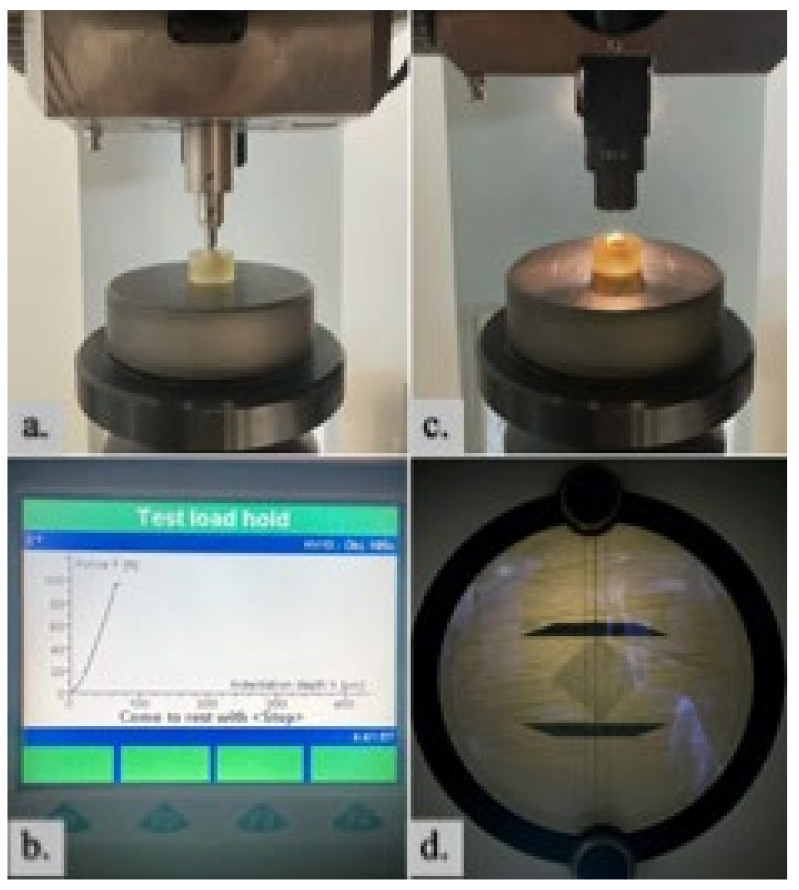
Vickers test procedure. (**a**) The position of the sample and indenter is determined; (**b**) The device mechanism is activated; (**c**) The microscope objective is adjusted; (**d**) The indentation size is measured.

The prepared and grouped samples were initially measured using the Zwick/Roell universal hardness tester “ZHU250” (Zwick GmbH & Co. KG, Ulm, Germany). The measurements were performed with a pyramidal diamond-tipped indenter, featuring an apex angle of 136° between opposite planes. The Vickers hardness testing method was chosen, utilizing the HV10 scale with a 100 N force. During the test, a 10 s load of 10 kg was applied, and measurements were conducted under 185× optical magnification. All measurements were performed by the same operator to ensure consistency. Each sample was measured three times at different surface points, and the arithmetic mean of the obtained measurements was calculated. The surface hardness measurement process is presented in Figure 4.

### 2.6. Phase of Artificial Enamel Lesions

**Figure 5:**
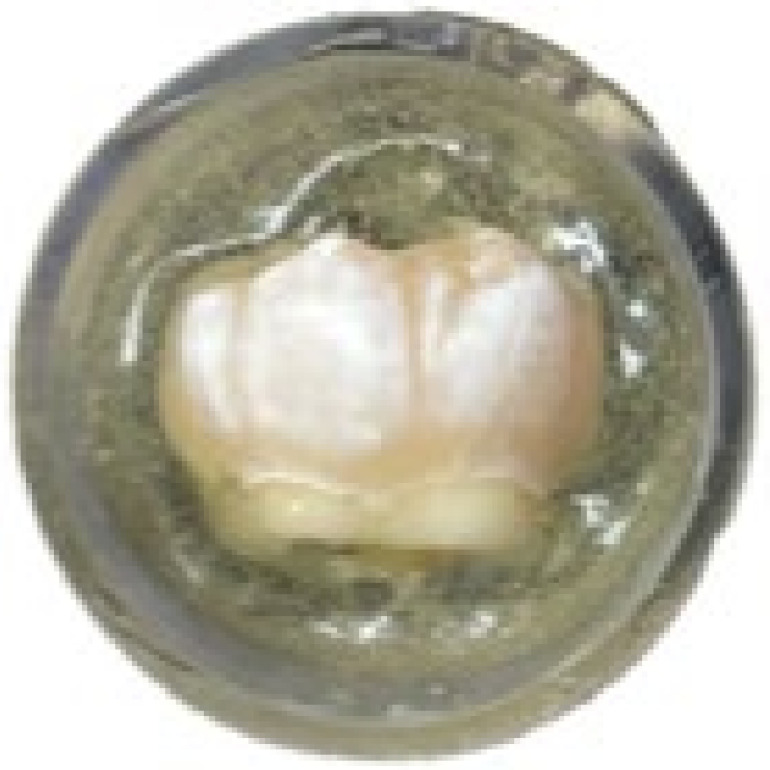
Tooth sample after demineralization.

A demineralizing acetic acid buffer solution was used to create surface lesions following the recommendations of Buskes and co-authors [29]. The solution was composed of CaCl_2_·2H_2_O (Riedel-de Haën, Sigma-Aldrich Laborchemikalien GmbH, Seelze, Germany), KH_2_PO_4_ (Riedel-de Haën, Sigma-Aldrich Laborchemikalien GmbH, Seelze, Germany), CH_3_COOH (Sigma-Aldrich, St. Louis, MO, USA), and MHDP. The pH value of the solution was adjusted to pH = 4.4 using KOH (Honeywell, Charlotte, NC, USA). Each sample was placed in a separate sterile container labeled with the group and sample number. The container with the sample was filled with the solution, and the containers were placed in a Thermo Scientific Heraeus^®^ incubator (Thermo Electron LED GmbH, Langenselbold, Germany) at a constant temperature of 37 °C. The samples were kept in the solution for 14 days. After removing the samples, the remaining solution was carefully rinsed with distilled water and air-dried for surface control. The tooth surface exhibited a matte, white appearance characteristic of typical early-stage enamel lesions (Figure 5). In the artificial lesions phase, the second surface hardness measurement of the samples was performed.

### 2.7. Application of Remineralizing Agents

According to the protocol, the following treatments were applied to the artificially created enamel lesions:

CG: No additional materials were applied.

P11-4: “Curodont™ Repair” (Credentis AG, Windisch, Switzerland) was applied. As per the manufacturer’s recommendations, the enamel surface was first cleaned for 20 s using a 2% sodium hypochlorite (NaOCl) solution, followed by rinsing with distilled water for 30 s. A 35% phosphoric acid gel (“KETCHANT Syringe”, Kuraray Co., Ltd., Tokyo, Japan) was then used to etch the enamel surface for 30 s, followed by another 30 s rinse with distilled water. “Curodont™ Repair” was dissolved in 0.05 mL of distilled water and applied to the etched surface. The material was left for 5 min to allow full penetration into the lesion.

FV: A fluoride varnish containing 22,600 ppm of 5% NaF (“Flairesse Varnish”, DMG Chemisch-Pharmazeutische Fabrik GmbH, Hamburg, Germany) was used. Before application, the varnish was thoroughly mixed and applied as a thin, uniform layer on the enamel surface. The varnish was left for 20 s and then air-dried.

Nano-HA: A 20% nano-hydroxyapatite varnish (“ApaCare Zahnlack”, ApaCare, Cumdente GmbH, Tübingen, Germany) was shaken before use and applied to the enamel surface using a microapplicator. The varnish was allowed to dry for 20 s.

All treatments were applied by the same dentist. After the application of the investigative materials, all samples were placed in an artificial saliva solution prepared according to the protocol by Buskes et al. [29]. The remineralization buffer solution was made using the following chemicals: calcium chloride dihydrate (CaCl_2_·2H_2_O, Riedel-de Haën, Sigma-Aldrich Laborchemikalien GmbH, Seelze, Germany), monopotassium phosphate (KH_2_PO_4_, Riedel-de Haën, Sigma-Aldrich Laborchemikalien GmbH, Seelze, Germany), and HEPES (Sigma-Aldrich, St. Louis, MO, USA). The solution’s pH was adjusted to 7.0 using potassium hydroxide (KOH, Honeywell, Charlotte, NC, USA). The samples were kept in the remineralizing buffer solution for 14 days. After removal, the samples were rinsed with distilled water. During the remineralization phase, the third enamel surface hardness measurement was conducted.

### 2.8. Statistical Data Analysis

Statistical data analysis was performed using the SPSS IBM 27.0 software (Statistical Package for Social Sciences). The normality of the distribution of quantitative variables was assessed using the Kolmogorov–Smirnov test. Due to the non-normal distribution of variables and the small sample size, non-parametric statistical methods were chosen. Comparisons of quantitative variables between experimental groups were conducted using the Kruskal–Wallis test, with Dunn’s test applied for multiple comparisons. The Wilcoxon signed-rank test was used for comparisons of dependent samples. A two-sample *t*-test was conducted to justify the study sample. Differences between variables were considered statistically significant when the *p*-value obtained from the applied test was less than 0.05.

## 3. Results

The enamel surface hardness results for each group during different phases are presented in Table 1.

**Figure 6:**
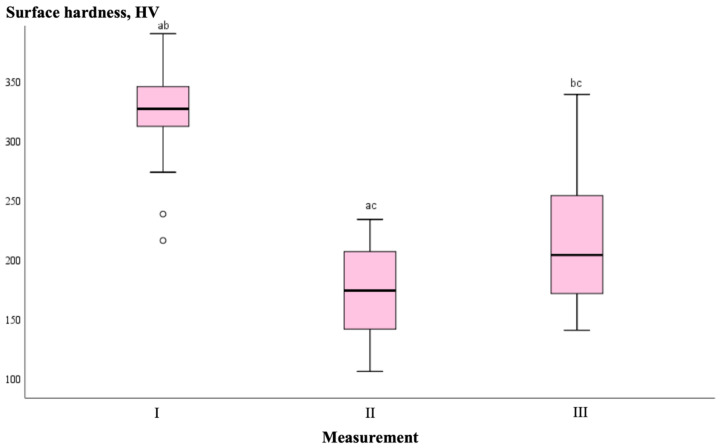
Boxplot of surface hardness for all samples at different measurement stages. Based on the non-parametric Wilcoxon test for dependent samples, a statistically significant difference was found between measurements (^abc^ *p* ≤ 0.001), irrespective of the experimental groups.

Based on the non-parametric Wilcoxon test, changes in enamel surface hardness for the entire sample (*n* = 36) between different measurement phases were assessed. The results showed statistically significant differences (*p* ≤ 0.001), indicating that both the demineralization and remineralization phases significantly altered the enamel surface hardness, irrespective of the experimental groups. The mean initial enamel surface hardness during the first measurement was 323.95. After the 14-day demineralization phase, resulting in artificial enamel lesions, the mean surface hardness value significantly decreased to 172.17. Following the 14-day remineralization phase, the mean surface hardness value significantly increased to 213.21 (*p* = 0.001). These changes are graphically represented in Figure 6.

**Figure 7:**
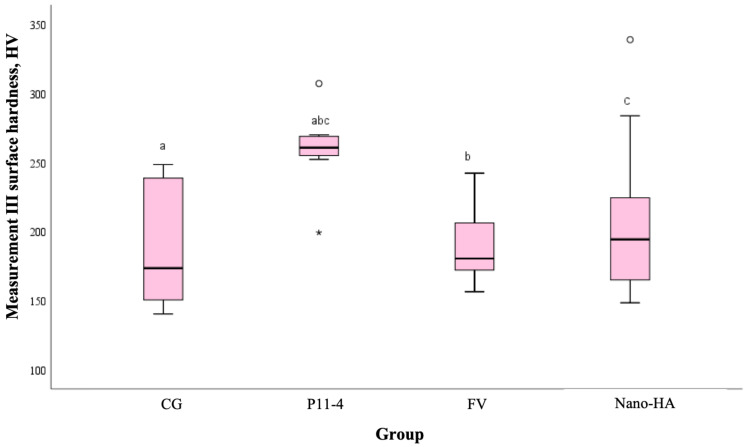
Boxplot of surface hardness for the third measurement, categorized by groups. Based on the non-parametric Kruskal Wallis test, a statistically significant difference was found between the P11-4 group (* *p*< 0.05) and all other groups (^abc^ *p* < 0.05). No statistical significance was observed among the FV group and nano-HA group compared to the control group (*p* > 0.05).

The statistical analysis using the non-parametric Kruskal–Wallis test revealed no significant differences in enamel surface hardness among the four groups in the first and the second measurements (*p* > 0.05) but showed significant differences between the groups only in the third measurement (*p* = 0.005). The control group (CG) had the lowest hardness values, while the P11-4 group exhibited the highest. Dunn’s test confirmed that the P11-4 group showed statistically significant superiority over all other groups (*p* < 0.05). However, the FV group and nano-HA group did not show significant differences compared to the control group (*p* > 0.05). The result is graphically represented in Figure 7.

The paired comparison analysis using the Wilcoxon test showed that enamel surface hardness significantly decreased after the demineralization phase compared to healthy enamel (I–II measurements, *p* < 0.01) and remained significantly lower even after remineralization (I–III measurements, *p* < 0.01). None of the materials restored enamel hardness to the original level. However, the P11-4 group demonstrated a statistically significant improvement in enamel hardness after remineralization (II–III measurements, *p* = 0.000), while the CG, FV, and nano-HA groups showed no significant changes (*p* > 0.05). The P11-4 group exhibited the greatest increase in surface hardness (87.30 HV), whereas the CG showed the smallest improvement (15.97 HV) (Table 2).

To determine the effectiveness of remineralizing materials on demineralized enamel surfaces, the percentage change in enamel surface hardness (Δ*HV*%) was calculated using this formula:

$$\Delta HV=\frac{HV_{III}-HV_{II}}{HV_{II}}\times 100\%$$

where:

*HV_II_*—change in enamel surface hardness after measurement II;

*HV_III_*—change in enamel surface hardness after measurement III.

**Figure 8:**
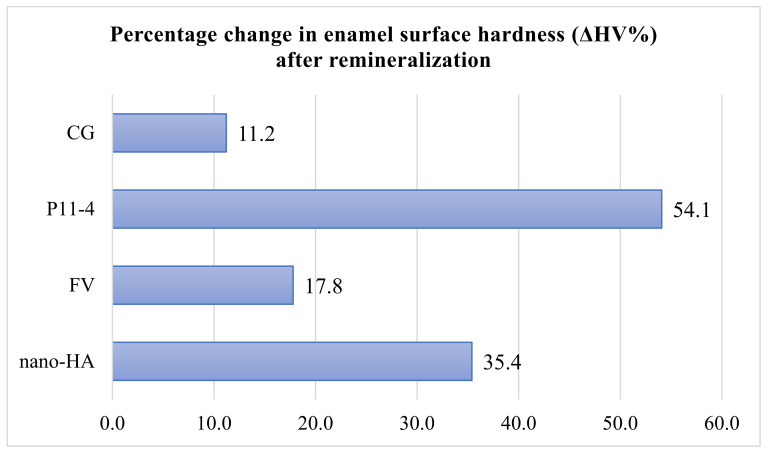
Percentage change in enamel surface hardness (Δ*HV*%) after remineralization.

The percentage change values for all groups are illustrated in Figure 8. The greatest percentage change in hardness was observed in the P11-4 group, which restored more than half of the enamel surface hardness lost during the demineralization phase (54.1%). The least improvement was seen in the CG, where the surface hardness increased by only 11.2% compared to the hardness after artificial enamel lesions were created. The second-best material was the 20% nano-HA varnish, which increased surface hardness by more than a third (35.4%) after the demineralization phase. Fluoride varnish performed slightly worse, restoring 17.8% of the previous enamel hardness.

## 4. Discussion

There is a widely accepted principle in modern dentistry that prioritizes preserving the natural tooth structure and restoring it only when necessary [30]. Conservative treatments have proven to be just as effective as traditional methods regarding clinical outcomes and durability. Additionally, conservative approaches are less time-consuming, cause less discomfort, and are more cost-efficient over time [31]. As a result, significant efforts are being made to halt the progression of dental caries. Although clinical trials remain the gold standard, standardized in vitro models are frequently used in cariology research and are essential for assessing the efficacy of remineralizing agents against dental caries [32].

Based on the findings of this study, the remineralization phase outcomes revealed statistically significant differences between groups. The peptide P11-4 was the most effective remineralizing material tested, demonstrating the highest percentage of enamel surface hardness restoration (54.1%). It was significantly more effective than the other tested materials, including the control, fluoride varnish, and nano-HA varnish. This superior performance is likely due to the peptide’s ability to facilitate biomimetic mineralization by forming hydroxyapatite crystals. In contrast, neither the fluoride varnish nor the nano-HA varnish produced statistically significant effects compared to the control group and no significant difference was found between these two materials. The control group showed the least remineralization since artificial saliva alone could not provide sufficient calcium and phosphate ions to match the effectiveness of other remineralizing therapies.

The differences in results can be attributed to the distinct mechanisms of action of the remineralizing agents. Fluoride varnish primarily strengthens the outer enamel layer, leaving the subsurface lesion soft and porous [12]. While fluorapatite formed during fluoride application is stronger and more stable, it cannot entirely replace hydroxyl groups, even in severe cases of enamel fluorosis [33]. Similarly, nano-HA varnish predominantly deposits minerals on the outer enamel surface, limiting calcium and phosphate ion penetration into deeper layers [34]. This results in only partial lesion recovery. In contrast, the self-assembling peptide P11-4 effectively restores subsurface lesions by forming beta-sheet assemblies that attract calcium and phosphate ions from surrounding fluids and reorganize them into a crystalline structure [35].

The conducted study confirmed the conclusions of other authors. The researchers state that the peptide P11-4 can induce biomimetic mineralization of early caries lesions in vitro [14,18,28,36,37]. As indicated by Soares et al. [38] and Tripathi et al. [39], in vitro studies showed that the peptide P11-4 significantly remineralized enamel lesions and demonstrated the best results among all study groups. Several researchers have also noted the superiority of peptide P11-4 over high-concentration fluoride varnish [19,24,40]. Krishnamoorthi et al. demonstrated that P11-4 significantly promoted the regeneration of enamel lesions, especially when used in combination with a fluoridated calcium-phosphate-based agent [41]. On the other hand, there are also studies in which peptide P11-4 did not show superiority over other materials [42,43].

The demineralization protocol used in this research, involving an acetic acid buffer solution, proved effective and corroborated previous findings [16,44]. This buffer significantly reduced enamel surface hardness, enabling a quantitative assessment of the remineralizing agents. The protocol’s uniqueness lies in the inclusion of MHDP, which inhibits enamel dissolution and promotes subsurface demineralization without affecting the surface, simulating early-stage caries lesions [45].

Surface hardness analysis was a reliable method for evaluating enamel changes during demineralization and remineralization. These findings support earlier studies on the effectiveness of remineralizing agents in improving enamel hardness [15,43].

Several factors may have influenced the results. Surface hardness measurements can be imprecise due to anatomical variations in tooth structure, including surface curvature and uneven enamel thickness. Furthermore, in vitro studies cannot fully replicate the oral environment, including biological factors such as saliva, enzymes, and plaque. The inorganic composition of the study solutions also ignores the impact of salivary proteins and pellicle on mineralization. Other limitations include experimental variability, differences in enamel microstructure, and the relatively short duration of the study.

Nevertheless, using artificial saliva and strict adherence to material application protocols ensured controlled conditions. The in vitro model also has notable advantages, such as allowing precise single-variable experiments in a highly controlled environment. This method is ideal for comparing various materials and understanding their mechanisms of action [46].

This study is one of the few to investigate the effects of the SAP P11-4 in vitro and the first to compare its performance against nano-HA varnish for artificial caries lesions. It is also the first research involving SAP P11-4 conducted in Lithuania.

In summary, despite its limitations, this in vitro study supports previous findings that the oligomeric peptide P11-4 significantly regenerates early enamel lesions. The material shows great promise for integration into routine dental practice as an effective tool for caries prevention. Further in vitro and in vivo studies comparing SAP P11-4 and nano-HA should be conducted to assess these materials over a long-term period.

## 5. Conclusions

The demineralizing solution significantly reduced enamel surface hardness, while peptide P11-4 effectively remineralized the lesions. Furthermore, the oligomeric peptide P11-4 proved to be significantly more effective in remineralizing artificial enamel lesions compared to fluoride varnish and nano-hydroxyapatite varnish. While many studies confirm the efficacy of peptide P11-4 in remineralizing early caries lesions in in vitro settings, future literature should focus on long-term clinical trials to assess its effectiveness in clinical applications and patient outcomes.

## Figures and Tables

**Table 1:** The enamel surface hardness results (HV) during different measurements across groups. A statistically significant difference was found between measurements (*p* ≤ 0.001), irrespective of the experimental groups.

Measurement No.	Group	*n*	Median Surface Hardness Values, HV	*p*-Value
I	CG	9	328.60	0.758
	P11-4	9	320.87	
	FV	9	331.03	
	Nano-HA	9	324.90	
	Total	36	326.55	
II	CG	9	174.83	0.211
	P11-4	9	163.47	
	FV	9	172.73	
	Nano-HA	9	178.93	
	Total	36	173.78	
III	CG	9	173.43	0.036
	P11-4	9	260.60	
	FV	9	180.40	
	Nano-HA	9	194.23	
	Total	36	203.57	

**Table 2:** Differences in surface hardness (HV) values across different phases. A statistically significant difference was found between the I–II and I–III measurements in all groups (*p* < 0.01) and between II–III measurements in the P11-4 group (*p* = 0.000). No statistical significance was observed between II–III measurements in the CG, FV, and nano-HA groups (*p* > 0.05).

Comparative Measurements	Group			
	1 (CG)	2 (P11-4)	3 (FV)	4 (Nano-HA)
	Average Difference (SD), HV			
I–II	150.00 (48.02) *	157.11 (40.62) *	149.17 (62.54) *	150.82 (33.45) *
I–III	134.03 (59.60) *	69.81 (43.58) *	127.99 (46.61) *	111.10 (74.28) *
II–III	15.97 (41.89)	87.30 (36.14) *	21.18 (52.32)	39.72 (92.85)

* Statistically significant differences (*p* < 0.01).

## Data Availability

Data are contained within the article.
